# Amiodarone-Induced Thyrotoxic Thyroiditis: A Diagnostic and Therapeutic Challenge

**DOI:** 10.1155/2014/231651

**Published:** 2014-11-12

**Authors:** Umang Barvalia, Barkha Amlani, Ram Pathak

**Affiliations:** ^1^Department of Internal Medicine, Marshfield Clinic, 1000 N. Oak Avenue, Marshfield, WI 54449, USA; ^2^Department of Endocrinology, Marshfield Clinic, Marshfield, WI 54449, USA

## Abstract

Amiodarone is an iodine-based, potent antiarrhythmic drug bearing a structural resemblance to thyroxine (T4). It is known to produce thyroid abnormalities ranging from abnormal thyroid function testing to overt hypothyroidism or hyperthyroidism. These adverse effects may occur in patients with or without preexisting thyroid disease. Amiodarone-induced thyrotoxicosis (AIT) is a clinically recognized condition commonly due to iodine-induced excessive synthesis of thyroid, also known as type 1 AIT. In rare instances, AIT is caused by amiodarone-induced inflammation of thyroid tissue, resulting in release of preformed thyroid hormones and a hyperthyroid state, known as type 2 AIT. Distinguishing between the two states is important, as both conditions have different treatment implications; however, a mixed presentation is not uncommon, posing diagnostic and treatment challenges. We describe a case of a patient with amiodarone-induced type 2 hyperthyroidism and review the current literature on the best practices for diagnostic and treatment approaches.

## 1. Introduction

Amiodarone is a potent antiarrhythmic drug used in the treatment of various ventricular and supraventricular arrhythmias. It carries a structural resemblance to thyroxine (T4) and can produce a wide spectrum of thyroid gland dysfunctions. Amiodarone-induced thyrotoxicosis (AIT) is commonly due to iodine-induced excessive synthesis of thyroid, also known as type 1 AIT (AIT1). However, in rare instances, AIT is due to amiodarone-induced inflammation of the thyroid tissue resulting in release of preformed thyroid hormones or a hyperthyroid state, known as type 2 AIT (AIT2). Due to incomplete understanding of the pathogenesis, unreliable response to therapy, and lack of a systemic approach, AIT2 continues to challenge the clinical acumen of internists and endocrinologists. Besides thyroid function testing, ultrasonography, radioiodine uptake and scan, interleukin-6 (IL-6) level determination, and color flow Doppler sonography (CFDS) aid in diagnosis and assessing the response of AIT2 to therapy. Use of 99mTc-sestamibi (MIBI) scanning is emerging as a beneficial adjunct to diagnosis. The current literature still supports steroids as the mainstay for treatment of AIT2. Iopanoic acid and perchlorate are reasonable therapeutic options but have not been shown to provide benefit when used with steroids. Close monitoring is essential, as the onset of AIT2 has been found to be a predictor of adverse cardiovascular outcomes [1].

## 2. Case Presentation

A 63-year-old man had been on amiodarone therapy 200 mg/day for approximately 2 years for recurrent angina pain secondary to paroxysmal atrial fibrillation despite control of ventricular rate. He was referred to the endocrinology department for concerns of hyperthyroidism diagnosed during routine follow-up of thyroid function monitoring related to his exposure to amiodarone. He reported a 19-pound weight loss in the past year, some of it intentional, and worsening of a hand tremor that he had for years. He denied any other symptoms of hyperthyroidism.

The past medical history was significant for nonocclusive coronary artery disease, hypertension, morbid obesity, and peripheral vascular disease with claudication. His family history included a sister with thyroid-related symptomology, but he was not able to provide further details. The patient was alert, in no acute distress, with a body weight of 263 pounds. Examination of the thyroid gland did not reveal palpable nodules on either lobe. The gland moved freely during swallowing and was somewhat diffusely enlarged. No lymphadenopathy or tenderness was noted on palpation. Vital signs were normal with a blood pressure of 110/60 mm Hg. The remainder of the examination was unremarkable.

**Figure 1:**
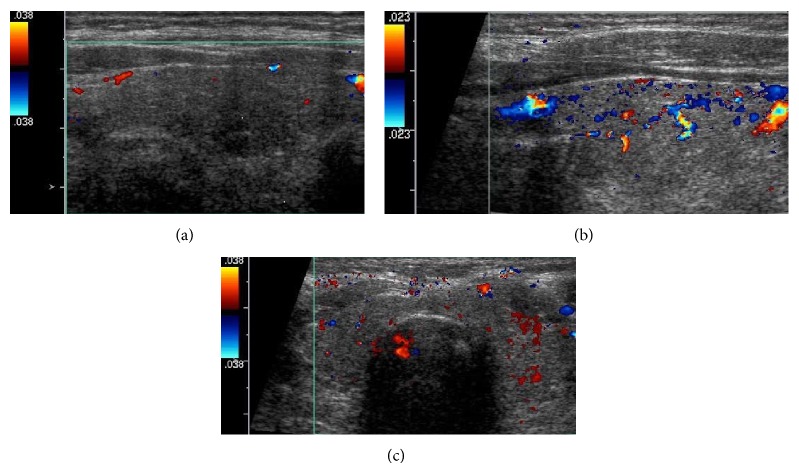
Thyroid ultrasound with color Doppler flow study showing normal to minimally reduced vascularity of the glandular tissue. (a) Long axis view of the right thyroid lobe. (b) Long axis view of the left thyroid lobe. (c) Transverse axis view of both lobes of thyroid gland including the isthmus.

**Figure 2:**
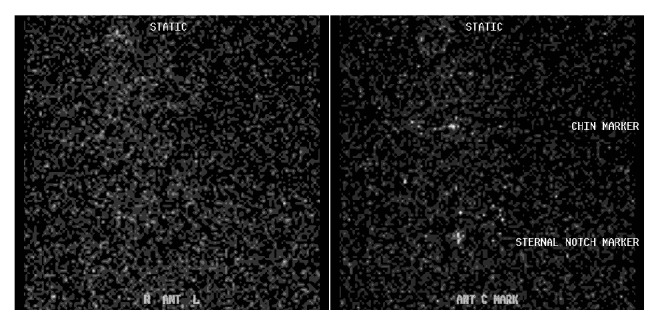
Radioiodine uptake and scan using 143 *μ*Ci of I-123 showing 0.6% uptake at 4 hours (no activity within the thyroid gland).

Thyroid function tests revealed a thyroid stimulating hormone (TSH) level of 0.01 mIU/mL (range 0.35–4.5 mIU/mL), free T4 of 2.7 ng/dL (range 0.6–1.2 ng/dL), and free T3 levels of 3.7 pg/mL (range 2.1–4.1 pg/mL). Thyroid ultrasound did not show hypervascularity (Figure 1). An ultrasound done 2 years previously had revealed a multinodular gland with a dominant right-sided 1.3 × 0.8 cm nodule that was found to be benign on fine needle aspiration biopsy. There was another 1.1 × 0.6 cm nodule on the left side that was not biopsied. The right-sided dominant nodule remained stable, and the left-side nodule had slightly increased to 1.2 cm in size. Radioiodine uptake and scan had 0.6% (range 4–20%) and 0.2% (range 5–30%) uptake at 4 hours and 24 hours, respectively, consistent with amiodarone-induced thyrotoxicosis in the clinical context (Figure 2). Screening for thyroglobulin antibody was normal at <20 IU/mL. The thyroglobulin level was 22.6 ng/mL, and IL-6 level was elevated at 8.63 pg/mL (range 0.31–5 pg/mL).

Although none of the findings were diagnostic, they favored a diagnosis of AIT2, more so than the more common AIT1. The patient was treated with low-dose steroids (20 mg prednisone daily) for a month with deescalation of dosing following improvement in thyroid function tests. He reported improvement in the bilateral hand tremor, back to his baseline, and was euthyroid with TSH of 3.09 mIU/mL, free T4 of 1.0 ng/dL, and free T3 of 2.5 pg/mL during subsequent follow-up 3 months later. The patient remained euthyroid clinically and biochemically without recurrence of AIT, while continuing amiodarone therapy for 3 years following treatment of the AIT2.

## 3. Discussion

Amiodarone is used in treating various atrial and ventricular arrhythmias and is favored in patients with left ventricular dysfunction [2]. Due to the structural similarity with thyroid hormones and the iodine content in the medication, it is known to cause significant changes in thyroid function testing, often with a clinically significant impact [3].

### 3.1. Mechanisms of Amiodarone-Related Thyroid Dysfunction

Amiodarone inhibits type 1 5′-deiodinase activity in the liver/peripheral tissues, which may persist for weeks following withdrawal of the drug [4]. Further, the drug inhibits type 2 5′-deiodinase activity in the pituitary, which reduces conversion of T4 to T3 and increased TSH levels [5]. Whereas amiodarone has no effect on the metabolism (distribution and removal) of plasma T3 pool [6], the drug does inhibit thyroid hormone entry into circulation [7].  Table 1 summarizes the effects of amiodarone on thyroid function tests in euthyroid patients.

Due to its intrinsic properties, amiodarone is associated more with hypothyroidism than thyrotoxicosis in iodine replete countries like the United Kingdom and the United States [8]. Pathogenesis of amiodarone-induced hyperthyroidism is still incompletely understood, which makes the diagnosis and treatment challenging. Two main types of AIT, with different mechanisms, have been described. AIT1 is due to iodine-induced excess synthesis and release of thyroid hormones, usually from abnormal thyroid glands. AIT2 is a form of destructive thyroiditis that leads to leakage of preformed hormones into the circulation. Though iodine excess may be an important pathogenic factor in both subtypes, some forms of AIT may be purely due to subacute thyroiditis and release of preformed hormones.

### 3.2. Diagnoses of AIT2

AIT usually has a sudden onset, presenting with a new or worsening arrhythmia, or it can be asymptomatic, especially in younger individuals [9, 10]. Because type 1 hyperthyroidism often occurs in patients with preexisting thyroid disease, the onset is within the first few months, whereas median time for occurrence of AIT2 is about 30 months following initiation of amiodarone therapy [11]. In either case, the initial evaluation should include the usual work-up for thyroid function including TSH, T3, T4, and antithyroid antibodies. Thyroid ultrasound with or without CFDS and radioiodine uptake (RAIU) and scan are useful in distinguishing the two AIT subtypes.

In AIT2, RAIU would be lower (<1%) compared to AIT1, where it is either normal or increased (>10%) [12]. In the United States, where most patients are iodine replete, RAIU can be low in both types of AIT, so CFDS is helpful in directing therapy [13, 14]. CFDS gives real time information on the blood flow inside the thyroid gland and its morphology. Due to follicular destruction, lymphocyte infiltration consequential to inflammatory responses seen in AIT2, the color flow on ultrasound would show increased vascularity and blood flow velocity [15]. These findings are indicative of a hyperfunctioning gland and are also seen in untreated Grave's disease. CFDS can serve to facilitate decision making due to its relative ease of use, ability to obtain faster results, and the noninvasive nature of the study, especially in patients with life-threatening tachyarrhythmias.

MIBI scanning has been used for detection of hyperfunctioning parathyroid adenomas and some malignant or benign thyroid tumors. There has been recent interest in its use in distinguishing between the two types of AIT. A small study of 20 patients found that it was superior to CFDS in differentiating between AIT1 and AIT2 [16].

Interleukin-6 (IL-6), a cytokine associated with inflammation, was proposed as a biomarker to distinguish between amiodarone-induced thyroiditis and iodine-induced hyperthyroidism. Marked elevations of IL-6 levels correlated closely with subacute thyroiditis in patients without preexisting thyroid disease. Normal to mild elevations of IL-6 were also found in patients with AIT1 [17].  Table 2 presents a comparative summary between the two types of amiodarone-induced hyperthyroidism.

### 3.3. Treatment

The best available treatment option for AIT2 is oral glucocorticoids. They act by reducing the inflammation in the thyroid gland, the primary pathologic mechanism in AIT2, and also reduce the peripheral conversion of T4 to T3 [18]. Baseline free T4 concentrations and thyroid gland volume can predict delayed responders to glucocorticoids. In individuals at high risk, this may help identify individuals in whom surgery and/or iopanoic acid should be considered early in the course of the disease [19].

Iopanoic acid, an oral cholecystographic agent (OCA), acts by inhibiting type 1 5′-deiodinase activity, the enzyme responsible for peripheral conversion of T4 to T3. A 70% reduction of serum T3 levels was observed after 48 h of iopanoic acid administration in spontaneous hyperthyroid patients, with little effect on serum T4 concentration. However, iopanoic acid does not affect the destructive thyroiditis processes associated with AIT2 [20]. Iopanoic acid is a reasonable alternative to steroids to control hyperthyroidism in the short term, but it would take longer than the use of steroids in achieving the euthyroid state, as shown in a small, prospective, randomized control trial [21].

Due to coexistence of both forms of AIT, there is often a variable response to glucocorticoid therapy in AIT2. Thionamides are sometimes needed in addition to glucocorticoids to achieve euthyroid status in these patients. IL-6 levels can aid in choosing therapy, but it may take days to weeks for the levels to be reported. Therefore, in severely ill patients, until the diagnosis is certain, it is prudent to treat both AIT1 and AIT2 with antithyroid drugs and glucocorticoids with or without perchlorate. Thyroidologists may employ a stepwise approach, where initial therapy would constitute using thionamides for 4 weeks and introduce steroids if there is insufficient or no response to thionamide therapy [22].

Guitierrez-Repiso et al. [23] observed that, in an adult population with adequate and stable nutrition, the iodine excretion in a random urine sample represented 70–80% of the daily iodine intake. This fact was also utilized by several investigators for either diagnosis or treatment of drug-induced thyrotoxicosis [24–26]. Erdoğan and colleagues suggested treating AIT with prednisone, potassium perchlorate, and titrating methimazole using urinary iodine excretion [27].

The majority of thyroidologists in North America and Europe did not recommend measuring urinary iodine for management of AIT. Urinary iodine excretion would not be an ideal marker of individual iodine nutrition because of the intraindividual variability considering changes in dietary iodine intake and thyroid function [23]. In cases of recurrent, refractory, or mixed forms of AIT where euthyroid status is not achieved with steroids alone, monitoring monthly levels and continuing steroids or adding methimazole until urine iodine concentration normalizes (<200 *μ*g/day) might be reasonable.

Amiodarone and its derivative desethylamiodarone have a long half-life of 40 and 57 days, respectively, due to the lipophilic nature of the drug and subsequent concentration in various tissues including adipose tissue [28]. Hence, withdrawal of the drug should not have immediate beneficial effects. It remains unclear whether amiodarone should be continued after diagnosis. There have been instances of amiodarone-induced coronary vasospasm and ischemic ventricular fibrillation related to hyperthyroid states [29]. Bogazzi et al. showed that the drug delays restoration of euthyroid status, and there are higher chances of recurrence when amiodarone is continued [20]. Euthyroid states were still achieved with continuation of amiodarone [30]. Consequently, decisions must be made on a case-by-case basis, and the drug should be withdrawn only if it is not too risky for the patient.

Measurement of iodine uptake and/or urine iodine excretion can be done to make sure that the iodine load has resolved. In surveys, after restoration of euthyroidism and withdrawal of amiodarone, thyroid ablation was selected less frequently by North American thyroidologists in AIT1, while an expectant strategy was shared by both North Americans and Europeans in AIT2 [31–33]. If amiodarone therapy needs to be reinitiated, prophylactic RAI therapy or thyroidectomy was recommended in AIT1; “wait and watch” strategy was still adopted for majority of the patients with AIT2, unless there was a relapse [31–33].

Subtotal or total thyroidectomy may be needed in cases of AIT that progresses despite aggressive medical therapy, especially in critically ill patients in whom discontinuing amiodarone can be life threatening. In patients with AIT and left ventricular systolic dysfunction, left ventricular ejection fraction improved when they underwent total thyroidectomy following failure to achieve euthyroid state with optimal medical treatment [34]. Several case reports have been published on treatment with lithium, plasmapheresis, and/or methimazole in refractory cases [35–37]. Perchlorate was hypothesized to ameliorate the cytotoxic side effects of amiodarone on thyrocytes and thereby help in restoration of euthyroid status when a mixed type of AIT was suspected. A multicenter randomized control in Netherlands showed that perchlorate, when used alone or in combination with prednisone, did not improve outcomes [30]. AIT patients generally have low RAIU values, and radioiodine ablation might not be feasible; however, an open study showed that it might be an option even in those patients [38]. Using recombinant TSH to allow radioiodine as a treatment modality has been proposed; however, subsequent elevation of thyroid hormone levels could potentially worsen underlying cardiac problems in these patients [39].

It is important to follow up patient's status with thyroid function testing, even after restoration to euthyroid state, as they may develop temporary or permanent hypothyroidism and may subsequently benefit from hormone replacement therapy [40].

## 4. Conclusion

Amiodarone-induced hyperthyroidism still remains a diagnostic challenge due to its incompletely understood pathogenesis, unreliable response to therapy, and lack of systemic approach. Steroids are the mainstay for treatment of AIT2. IL-6 levels and CFDS may be helpful in aiding with diagnosis and assessing response to therapy. Surgery should be considered on a case-by-case basis in patients with uncontrolled hyperthyroidism with conservative measures or in whom watchful medical management could have deleterious effects. In case of progressive deterioration, where restoration of euthyroid status is essential, a short course of iopanoic acid followed by surgical removal of the gland should be considered. Lithium, plasmapheresis, and radioactive treatment have been attempted, but there is currently limited evidence to recommend their use.

## Figures and Tables

**Table 1:** Effects of amiodarone on thyroid function tests in euthyroid patients.

Test	Duration of treatment	
	Subacute (<3 months)	Chronic (>3 months)
T4	Modest increase	Remains increased by up to 40% above baseline; may be in high reference range or moderately raised
T3	Decreased, usually to low reference range	Remains in low reference range or slightly low
TSH	Transient increase (up to 20 mU/L)	Normal, but there may be periods of high or low values
rT3	Increased	Increased

Used with permission from *Heart* (Newman et al. 1998 [3]).

**Table 2:** ^*^Clinical and pathologic features distinguishing type 1 and type 2 amiodarone-induced hyperthyroidism^†^.

	Type 1	Type 2
Underlying thyroid disease	Yes	No
Thyroid ultrasound	Diffuse or nodular goiter	Normal (hypoechoic) gland (small goiter)
CFDS	Increased vascularity	Normal to reduced vascularity
Thyroid RAIU	Low/normal/increased	Low/absent
MIBI	Thyroid retention	Absent uptake
Pathogenesis	Iodine-induced hyperthyroidism	Destructive thyroiditis
Spontaneous remission	No	Possible
Preferred treatment	Thionamides (plus perchlorate)	Glucocorticoids
Posttherapy hypothyroidism	Unlikely	Possible

^*^Modified from Table 1, Bogazzi et al. [12].

^†^Mixed forms of AIT have not been fully understood and are believed to be a combination of iodine-induced hyperthyroidism and destructive thyroiditis from the drug itself.

CFDS: color flow Doppler sonography; RAIU: radioiodine uptake; MIBI: 99mTc-sestamibi.
